# Identification of the Role of miR-142-5p in Alzheimer’s Disease by Comparative Bioinformatics and Cellular Analysis

**DOI:** 10.3389/fnmol.2017.00227

**Published:** 2017-07-18

**Authors:** Juhyun Song, Young-Kook Kim

**Affiliations:** ^1^Department of Biomedical Sciences, Center for Creative Biomedical Scientists at Chonnam National University Gwangju, South Korea; ^2^Department of Biochemistry, Chonnam National University Medical School Jeollanam-do, South Korea

**Keywords:** miR-142-5p, Alzheimer’s disease (AD), synaptic plasticity, SH-SY5Y cells, postsynaptic density protein 95 (PSD-95)

## Abstract

Alzheimer’s disease (AD) is the most common neurodegenerative disease characterized by the formation of amyloid beta (Aβ) or tau protein aggregates, the hallmark of cognitive decline. MicroRNAs (miRNAs) have emerged as critical factors in neurogenesis and synaptic functions in the central nervous system (CNS). Recent studies have reported alterations in miRNA expression in patients with AD. However, miRNAs associated with AD varied with patient groups or experimental models, suggesting the need for a comparative study to identify miRNAs commonly dysregulated in diverse AD models. Here, we investigated the miRNAs that show dysregulated expression in two different human AD groups and mouse and cellular AD models. After selection of commonly dysregulated miRNAs in these groups, we investigated the pathophysiological significance of miR-142-5p in SH-SY5Y neuronal cells. We found that miR-142-5p was increased upon treatment with Aβ peptide 1–42 (Aβ_42_). Inhibition of miR-142-5p rescued the Aβ_42_-mediated synaptic dysfunctions, as indicated by the expression of postsynaptic density protein 95 (PSD-95). Among genes with decreased expression in Aβ_42_-treated SH-SY5Y cells, the predicted miR-142-5p target genes were significantly related with neuronal function and synapse plasticity. These findings suggest that dysregulation in miR-142-5p expression contributes the pathogenesis of AD by triggering synaptic dysfunction associated with Aβ_42_-mediated pathophysiology.

## Introduction

MicroRNAs (miRNAs) are non-coding RNA molecules that comprise approximately 22 nucleotides and suppress the translation of mRNAs by binding to their 3′-untranslated region (UTR; Davis et al., 2015; Kim, 2015; Cao et al., 2016; Reddy et al., 2017). As miRNAs regulate a variety of cellular processes, modulation of miRNA expression is considered a promising therapeutic approach for the diagnosis and treatment of diverse central nervous system (CNS) diseases (Gascon and Gao, 2012; Grasso et al., 2014; Qiu et al., 2015; Martinez and Peplow, 2016). Several studies demonstrated that over 70% of identified miRNAs are found in the brain (Shao et al., 2010). miRNAs are shown to control a variety of cellular mechanisms such as development, neuron survival, synaptic plasticity and secretion of neurotransmitter in the CNS (Cohen et al., 2011). Aberrations in the expression of miRNAs in the CNS contribute to neurodegenerative disorders, including Alzheimer’s, Parkinson’s and Huntington’s diseases (Cogswell et al., 2008; Saba and Schratt, 2010; Gascon and Gao, 2012; Salta and De Strooper, 2012; Grasso et al., 2014). Alzheimer’s disease (AD) is the most common neurodegenerative disease characterized by the formation of amyloid plaques, which are associated with neuronal loss and cognitive decline (O’Brien and Wong, 2011; Reddy et al., 2012). Studies have reported that the cognitive deficit in AD results from impaired synaptic transmission and plasticity by aberrant amyloid beta peptide 1–42 (Aβ_42_; Jo et al., 2011; Sheng et al., 2012). Clinical studies have reported an alteration in the miRNA expression in AD patients compared to normal subjects, which contributes to disease progression (Cogswell et al., 2008; Tan et al., 2014; Denk et al., 2015). Although these studies identified several miRNA candidates as biomarkers in AD, the detailed functional studies on miRNAs that contribute to the pathogenesis of AD, such as synaptic dysfunctions and cognitive decline, are largely unknown.

In the present study, we investigated miRNAs that show dysregulated expression in AD. We evaluated alterations in miRNA expressions in both the human and the mouse AD brain through comparative bioinformatics analysis. We selected the target candidate miRNA in the AD brain and analyzed its role in Aβ_42_-treated SH-SY5Y neuronal cells *in vitro*. Through the analysis of target genes of miRNA, we suggest the importance of miRNA in AD pathogenesis. Thus, we highlight that modulation of a specific miRNA in the AD brain is necessary to improve AD pathogenesis.

## Materials and Methods

### Analysis of miRNA Expression from Sequencing Data

**Figure 1:**
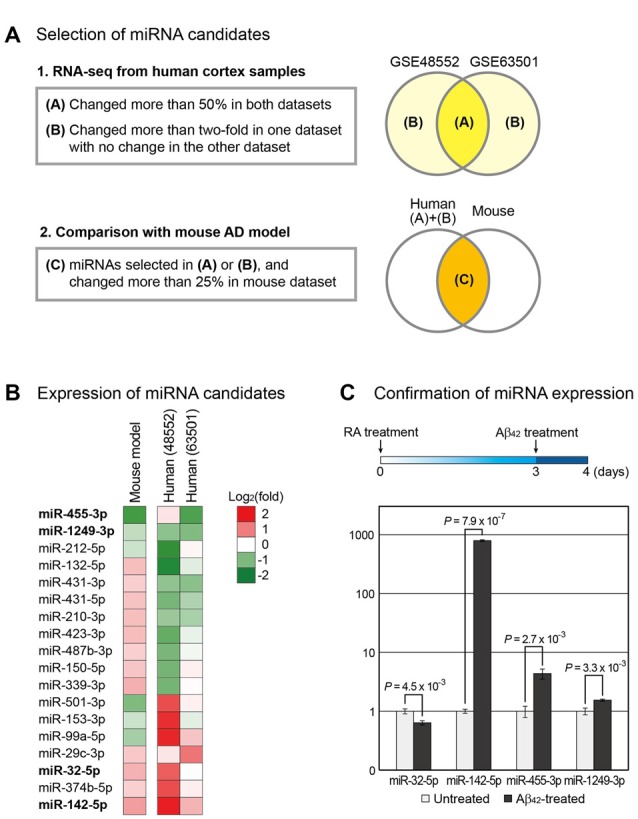
Selection of microRNA (miRNA) candidates associated with Alzheimer’s disease (AD). **(A)** Using small RNA sequencing data, miRNAs involved in the progression of AD were selected. Two independent data for human AD samples (GSE48552 and GSE63501) were obtained from the Gene Expression Omnibus (GEO) database (Lau et al., 2013; Santa-Maria et al., 2015). miRNAs that changed expression in both groups or exhibited highly altered expression in one group with no change in the other group were selected. Of these miRNAs, those with altered expression in the mouse AD model (GSE55589 dataset) were shortlisted (Luo et al., 2014). **(B)** Changes in the expression of miRNAs selected in **(A)** were compared between the AD and normal groups and shown by heat map. **(C)** To reconfirm the expression change of miRNAs in a cellular AD model, we differentiated SH-SY5Y cells into neurons, followed by their treatment with amyloid beta peptide 1–42 (Aβ_42_). The change in the expression of miRNAs was measured. The expression level of U6 snRNA was used as a normalization control. Error bars indicate standard error (*n* = 3). *P* values were calculated using two-tailed *t*-test.

We identified miRNAs with altered expression in AD models using raw data from small RNA sequencing obtained from Gene Expression Omnibus (GEO; GSE48552, GSE63501 and GSE55589)^1^. After deletion of the adaptor sequences, sequences with low quality were eliminated using FASTX-Toolkit^2^ and the remaining sequences were aligned to the human reference genome (GRCh38) by Bowtie2 (Langmead and Salzberg, 2012). Only the reads perfectly aligned to the human genome were selected. Genomic coordinates of miRNAs (obtained from http://www.mirbase.org/) modified with manual curation were used to calculate the miRNA counts (Kim et al., 2016). Using an in-house Perl script, we counted the sequencing reads corresponding to miRNA loci and normalized them by scaling whole read counts as one million (RPM, reads per million). miRNAs with average RPM values higher than 5 in each dataset were used for further analyses (Figure 1A).

### Cell Culture and Transfection

The SH-SY5Y neuroblastoma cell line was used in this study. SH-SY5Y cells are capable of differentiating into neuron-like cells in presence of retinoic acid (RA; Encinas et al., 1999; Xun et al., 2012; Shipley et al., 2016). Undifferentiated SH-SY5Y cells were cultured in Dulbecco’s Modified Eagle’s Medium (DMEM) supplemented with 10% fetal bovine serum (FBS; Gibco, Grand Island, NY, USA) and 100 μg/mL penicillin-streptomycin (Gibco, Grand Island, NY, USA) at 2 × 10^5^ cells/mL. After 2 days, the medium was replaced with DMEM supplemented with 10% FBS and 5 μM RA for neuronal differentiation. SH-SY5Y cells were cultured in a humidified atmosphere of 5% CO_2_ at 37°C. Synthetic miR-142-5p and control inhibitor were purchased from Ambion (Ambion, Austin, TX, USA). This inhibitor specifically binds to and blocks the function of endogenous miRNAs. SH-SY5Y cells were cultured for 3 days, followed by inhibitor treatment using Lipofectamine 3000 reagent (Thermo Fisher Scientific, Carlsbad, CA, USA) and incubation for one and a half days. One day before sampling, cells were treated with 10 μM Aβ_42_.

### Aβ_42_ Oligomer Preparation

Oligomeric Aβ_42_ was prepared as previously described (An et al., 2013). Briefly, 1 mg of synthetic Aβ_42_ peptide (American Peptide, Sunnyvale, CA, USA) was dissolved into 1 mL 1,1,1,3,3,3-hexafluoro-2-propanol (HFIP; Sigma Aldrich, St. Louis, MO, USA). The solution was dried under a nitrogen stream and resuspended in 100% HFIP at a concentration of 1 mg/mL. The solution was sonicated in a bath sonicator for 5 min and dried under nitrogen stream. The solution was re-dissolved in 1 mL HFIP and dried for 2 h. The film was resuspended in 200 μL dimethyl sulfoxide (DMSO; Sigma Aldrich, St. Louis, MO, USA) to obtain a 1 mM Aβ_42_ stock solution. For Aβ_42_ treatment, 10 μL of 1 mM Aβ_42_ was diluted with 1 mL DMEM media (final concentration, 10 μM) and incubated for 12 h at 37°C.

### Measurement of miRNA Levels

After removing culture media, SH-SY5Y cells were collected using cell scraper (Sigma-Aldrich, St. Louis, MO, USA). After centrifuging at 12,000 *g* for 3 min at 4°C, we used only cell pellets for RNA isolation. Total RNA in SH-SY5Y cells was extracted using TRIzol reagent (Thermo Fisher Scientific, Carlsbad, CA, USA). The concentration of RNA was measured by NanoDrop spectrophotometer (ND-1000, NanoDrop technology). Quantitative analysis of miR-32-5p, miR-142-5p, miR-455-3p and miR-1249-3p expression was performed with reverse transcription polymerase chain reaction (RT-PCR) using the TaqMan MicroRNA Reverse Transcription kit (Applied Biosystems, Waltham, MA, USA) and 10 ng of total RNA. PCR reactions were performed following the manufacturer’s guideline to quantitate the level of miR-32-5p, miR-142-5p, miR-455-3p and miR-1249-3p using TaqMan Universal PCR Master Mix, No Amp Erase UNG (Applied Biosystems, Waltham, MA, USA), and TaqMan MicroRNA assay (Applied Biosystems, Waltham, MA, USA). PCR amplification was conducted in Takara Real Time PCR (Takara, Shiga, Japan). Levels of miRNAs were presented as relative quantities (RQ) normalized to the level of U6 snRNA.

### Western Blot Analysis

For western blot analysis, SH-SY5Y cells were cultured in 100 mm plates. After treating the cells with each chemical, we harvested them using cell scraper (Sigma-Aldrich, St. Louis, MO, USA). After centrifuging at 12,000 *g* for 3 min, the supernatants were removed. The cell pellets were rinsed with phosphate-buffered saline (PBS) and collected by centrifugation again. SH-SY5Y cell pellets were lysed with ice-cold radioimmunoprecipitation assay (RIPA) buffer (Sigma-Aldrich, St. Louis, MO, USA). Cell lysates were centrifuged at 12,000 *g* for 30 min at 4°C to produce whole-cell extracts. Proteins (30 μg) were separated on a 10% sodium dodecyl sulfate (SDS)-polyacrylamide gel and transferred onto a polyvinylidene difluoride membrane. The membrane was blocked with skim milk prepared in TBS-T (20 nM Tris pH 7.2, 150 mM sodium chloride [NaCl], 0.1% Tween 20) for 90 min at room temperature, followed by incubation with primary antibody against amyloid precursor protein (APP; 1:1000; Abcam, Cambridge, MA, USA), Aβ_42_ (1:1000; Abcam, Cambridge, MA, USA) or β-actin (1:1000; Millipore, Billerica, MA, USA) for 18 h at 4°C. The membrane was then probed with a secondary antibody (Abcam, Cambridge, MA, USA) for 90 min at room temperature and visualized using enhanced chemiluminescence (ECL) solution (Millipore, Billerica, MA, USA).

### Immunocytochemistry

SH-SY5Y cells were washed thrice with PBS and fixed for 30 min with 4% paraformaldehyde solution. Cells were incubated with anti-rabbit postsynaptic density protein 95 (PSD-95; 1:500; Cell Signaling, Danvers, MA, USA) antibody for 18 h at 4°C. Following incubation, cells were washed twice with PBS and incubated with a secondary antibody for 90 min at room temperature. Cells were counterstained with 1 μg/mL of 4′,6-diamidino-2-phenylindole (DAPI, 1:100; Thermo Fisher Scientific, Carlsbad, CA, USA) for 15 min at room temperature. Images were obtained with a confocal microscope (Carl Zeiss, Thornwood, NY, USA) and the intensity of PSD-95 signal was measured using ImageJ software.

### Measurement of mRNA Levels

The amount of PSD-95 mRNA in SH-SY5Y cells was evaluated with quantitative real-time PCR. Total cellular RNA was extracted from SH-SY5Y cells using TRIzol reagent (Thermo Fisher Scientific, Carlsbad, CA, USA) and complementary DNA (cDNA) was synthesized using AccuPower Rocketscript Cycle RT Premix (Bioneer, Daejeon, Korea). The cDNA was mixed with SYBR premix EX-Taq RT-PCR Kit (Takara, Shiga, Japan) and specific primers in a total reaction volume of 20 μL. PCR was performed using the following primers: PSD-95 forward: 5′-TCGGTGACGACCCATCCAT-3′, PSD-95 reverse: 5′-GCACGTCCACTTCATTTACAAAC-3′; glyceraldehyde 3-phosphate dehydrogenase (GAPDH) forward: 5′-ACAACTTTGGTATCGTGGAAGG-3′, GAPDH reverse: 5′-GCCATCACGCCACAGTTTC-3′. GAPDH was used as an internal control. The ΔCt values from Aβ_42_-treated SH-SY5Y cells were compared with those from untreated SH-SY5Y cells.

### Analysis of miRNA Targets

We used the predicted target lists from TargetScan database^3^ to identify the target mRNAs of miR-142-5p (Agarwal et al., 2015). Only the transcripts with conserved targets sites corresponding to 947 genes were used. As the expression of miR-142-5p is greatly increased in Aβ_42_-treated cells (Figure 1), we selected mRNAs that exhibited decreased expression under same condition, using the microarray data for Aβ_42_-treated SH-SY5Y cells (GSE23000; Gatta et al., 2011). mRNAs from Aβ_42_-treated cells with expression below 0.8-fold compared to those from untreated cells were selected. These mRNAs were intersected with the list of predicted target genes of miR-142-5p. Finally, 102 genes were selected and subjected to gene ontology (GO) analysis at Molecular Signatures Database (Subramanian et al., 2005)^4^. The top 10 most significant GO terms were selected.

## Results

### Selection of miRNA Candidates Associated with AD

miRNAs commonly dysregulated in the brain of AD patients and mouse model were identified using the high-throughput sequencing data from GEO. GSE48552 and GSE63501 datasets were selected to obtain small RNA sequencing data from human cortex samples. The GSE48552 dataset was obtained from the analysis of the expression of small RNAs from six early-stage (control) and six late-stage AD patients (Lau et al., 2013). The GSE63501 dataset contained the small RNA sequencing data for brains from seven control and six AD patients (Santa-Maria et al., 2015). Using these two datasets, we selected miRNAs with altered expression in both datasets (more than 50%) or considerable change (more than two-fold) in either dataset as candidates involved in the pathogenesis of AD (Figure 1A and Supplementary Table S1).

We included the small RNA expression data from the mouse model of AD owing to the possible high variation in human data. We used GSE55589 dataset, which profiled small RNAs from the cortex of APP/PSEN1ΔE9 transgenic mouse and its sibling wild-type pair (Luo et al., 2014). This mouse model expresses mutant forms of APP and presenilin 1 (PSEN1) in neurons; both these proteins are associated with early-onset AD in humans. We compared the miRNA expression levels in this dataset to those selected from human datasets and selected only the miRNAs that displayed more than 25% change in mice (Figure 1A and Supplementary Table S1). Finally, we selected 18 miRNAs from these analyses (Figure 1B).

From the selected list, we chose miRNAs that showed either increased or decreased expression in both humans and mice. Moreover, candidates with other paralogous miRNA in the human genome were excluded to avoid complications in the analysis. Thus, we shortlisted four miRNAs—miR-32-5p, miR-142-5p, miR-455-3p and miR-1249-3p.

We reconfirmed the expression change of these miRNAs using the cell-based model for AD. A human neuroblastoma cell line SH-SY5Y that differentiates into neuronal phenotype on treatment with RA was used (Encinas et al., 1999; Xun et al., 2012; Shipley et al., 2016). After incubation with RA for 3 days, cells were treated with Aβ_42_. Total RNA was extracted the following day and the miRNA level was evaluated by quantitative PCR. We found that miR-142-5p was significantly increased in Aβ_42_-treated cells compared to control, consistent with the brain sample data from both humans and mice (Figure 1C and Supplementary Figure S1A). As a control for Aβ_42_ treatment, we measured the protein level of APP and found that its level evidently increased in Aβ_42_-treated cells (Supplementary Figure S1B). We selected miR-142-5p as the final candidate involved in the pathogenesis of AD.

### miR-142-5p Suppression Inhibits the Loss of PSD-95 in SH-SY5Y Neuronal Cells Treated with Aβ_42_

**Figure 2:**
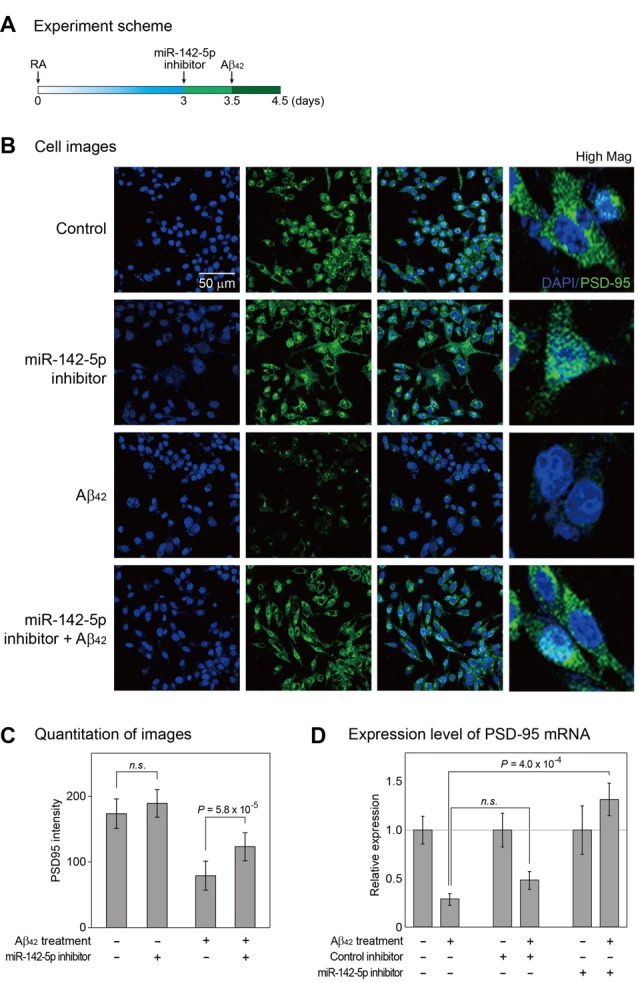
Cellular analysis to identify the role of miR-142-5p in Aβ_42_-treated SH-SY5Y cells. **(A)** Following incubation with retinoic acid (RA) for 3 days, SH-SY5Y cells were treated with miR-142-5p inhibitor for 1.5 days. After 12 h, cells were exposed to the Aβ_42_. **(B)** The expression of postsynaptic density protein 95 (PSD-95) was assessed by immunocytochemistry. Control indicates no treatment. High Mag: high magnification, 4′,6-diamidino-2-phenylindole (DAPI): blue signal, PSD-95: green signal. **(C)** The quantitation graph of immunointensity signal for PSD-95 in **(B)**. The result indicates high PSD-95 signal for cells pretreated with miR-142-5p inhibitor. *P* values were calculated by Wilcoxon rank-sum test with Bonferroni correction. **(D)** The mRNA level of PSD-95 was measured by quantitative real-time polymerase chain reaction. The mRNA level of PSD-95 increased in Aβ_42_-treated SH-SY5Y cells subjected to miR-142-5p inhibitor pretreatment. Data are presented as mean ± standard error (*n* = 4–6). *P* values were calculated using two-tailed *t*-test. Glyceraldehyde 3-phosphate dehydrogenase (GAPDH) was used as a normalization control. n.s. indicates not significant.

Given the increase in miR-142-5p expression during the pathogenesis of AD, we hypothesized that miR-142-5p suppression may alleviate the AD phenotype. We pretreated differentiated SH-SY5Y cells with miR-142-5p inhibitor, followed by treatment with Aβ_42_ (Figure 2A and Supplementary Figure S2). The effect of miR-142-5p inhibition in Aβ_42_-treated SH-SY5Y cells was investigated with the evaluation of PSD-95 expression. A loss of synaptic integrity leads to cognitive impairment in the AD brain (Coleman and Yao, 2003; Gylys et al., 2004), and PSD-95 as a neuronal scaffolding protein (Niethammer et al., 1996) is known to influence synapse maturation and regulate synaptic plasticity in the neuronal network (Kennedy, 2000; Elias et al., 2006). Several studies demonstrated that the level of PSD-95 was considerably reduced in the AD brain (Gylys et al., 2004; Love et al., 2006). Immunocytochemistry experiment revealed that the intensity of PSD-95 protein in SH-SY5Y cells was reduced after Aβ_42_ treatment as expected (Figures 2B,C; Leuba et al., 2008; Singh et al., 2017). However, pretreatment of SH-SY5Y cells with miR-142-5p inhibitor compromised the reduction in PSD-95 after Aβ_42_ treatment. This result was verified with the measurement of PSD-95 mRNA level (Figure 2D). Pretreatment with miR-142-5p inhibitor attenuated the decrease of PSD-95 mRNA expression after treatment with Aβ_42_. Taken together, our results show that the inhibition of miR-142-5p prevented the Aβ_42_-induced loss of PSD-95 in SH-SY5Y cells.

### Target Analysis of miR-142-5p and Its Functional Implication

**Figure 3:**
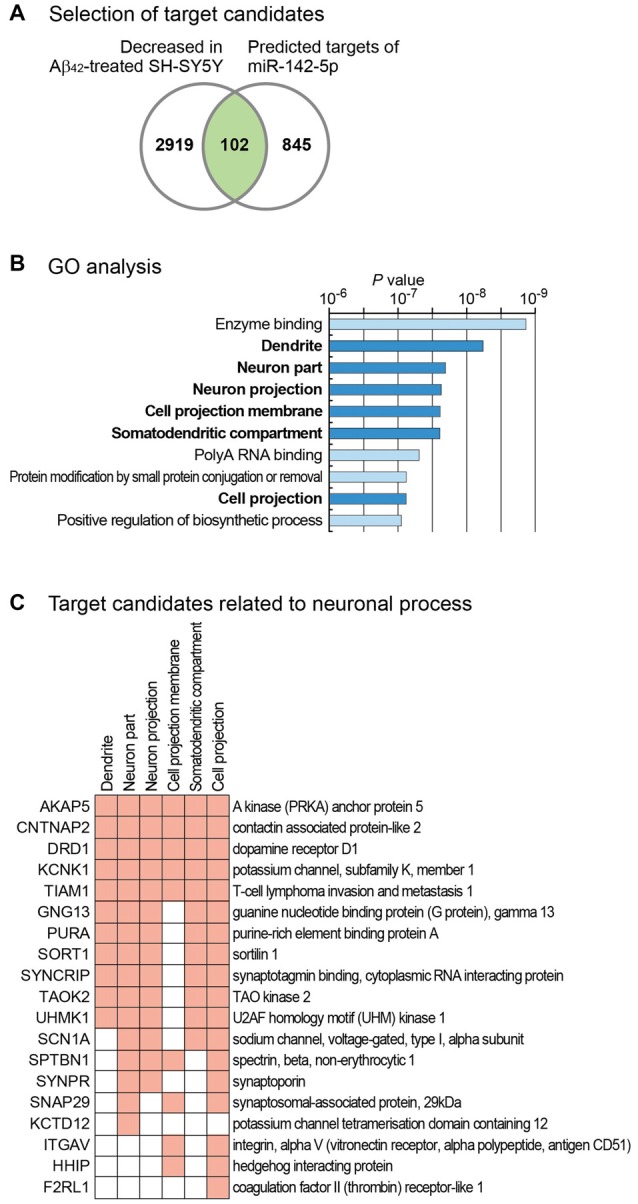
Target analysis of miR-142-5p and its functional implication. **(A)** For the identification of the target genes of miR-142-5p, we analyzed mRNA expression profile in SH-SY5Y cells after treatment with Aβ_42_ (GSE23000; Gatta et al., 2011). Only genes that exhibited decreased expression after Aβ_42_ treatment were chosen. We filtered these genes based on the existence of conserved target sites of miR-142-5p using predicted target list from TargetScan database (Agarwal et al., 2015). **(B)** Using the selected gene set in **(A)**, statistically enriched Gene Ontology (GO) terms were identified using Molecular Signatures Database (Subramanian et al., 2005). *P* values, which were calculated by hypergeometric test for each GO term, were obtained from the database. The terms related with neuronal process are shown with bold letters. **(C)** Target gene lists of miR-142-5p related with neuronal processes are shown. Among the target genes of miR-142-5p from **(A)**, those genes with neuronal process-related GO terms in **(B)** were chosen. Genes with corresponding GO terms are shown with filled boxes.

Our study shows that miR-142-5p plays a role in neuronal process. To identify target genes of miR-142-5p, we used the microarray data and measured global mRNA level changes after treatment of SH-SY5Y cells with Aβ_42_ (Gatta et al., 2011). Because miR-142-5p expression is highly increased by Aβ_42_ treatment, we selected only the mRNAs with decreased expression following Aβ_42_ treatment. In comparison to untreated controls, cells treated with Aβ_42_ showed a decrease below 0.8-fold in the expression of 3021 mRNAs (Figure 3A). As miRNAs regulate the expression of mRNAs by binding to the sequence-specific target sites on mRNAs, we intersected these selected genes with the predicted target list of miR-142-5p from TargetScan database (Agarwal et al., 2015). Of 947 genes with conserved target site(s), 102 genes showed decreased expression in Aβ_42_-treated SH-SY5Y cells (Figure 3A and Supplementary Table S2).

We analyzed the function of these genes by performing gene set analysis. GO analyses identified six neuron-related terms among the top 10 significantly enriched GO terms (Figure 3B). Of 102 selected genes, those with neuron-related GO terms are listed in Figure 3C. GO analysis for the decreased gene group with no target site for miR-142-5p (2919 genes) showed no neuron-related term among the top 10 enriched terms (data not shown). This analysis suggests that miR-142-5p plays a specific role in neuronal process during AD progression by targeting neuron-related genes.

## Discussion

AD is characterized by progressive impairment of synaptic plasticity, resulting in dysfunction of learning and memory processes (Pozueta et al., 2013). Previous studies identified several miRNAs that regulate the expression of those genes involved in the pathogenesis of AD. Beta-secretase 1 (BACE-1) and APP, which play important roles in AD, were identified as the targets of miR-135a and miR-200b, respectively (Liu et al., 2014). Another study revealed that miR-200c, which suppresses phosphatase and tensin homolog (PTEN), has a protective role in AD pathologies (Wu et al., 2016). These and many other studies showed that miRNAs have important roles in the progression of AD. In the present study, we added a new miRNA to this list, which could be a potential therapeutic target for the treatment of AD. We found that miR-142-5p suppression prevents the reduction of PSD-95 level in Aβ_42_-treated SH-SY5Y neuronal cells. In addition, bioinformatics analysis revealed the regulation of genes associated with neuronal maintenance and synaptic plasticity by miR-142-5p. As abnormal synaptic process is an important factor in cognitive dysfunction, our results suggest that miR-142-5p may be a potential target in AD for the improvement in synaptic signaling.

PSD-95, a major scaffold postsynaptic protein, affects the functional integrity of excitatory synapses (El-Husseini Ael et al., 2002; Blanpied et al., 2008). Previous studies showed a decrease in PSD-95 level in brains of AD patients (Gylys et al., 2004; Proctor et al., 2010), leading to neuronal death and memory deficits (Oakley et al., 2006; Leuba et al., 2008; Proctor et al., 2010). PSD-95 was identified as a target of miR-125-5p (Muddashetty et al., 2011). However, miR-125-5p expression showed no change in human AD samples and exhibited a slight decrease in the mouse AD model (data not shown) and hence, may not be relevant to the pathogenesis of AD. Although the decrease in PSD-95 expression after Aβ_42_ treatment was compromised through the inhibition of miR-142-5p, the underlying mechanism is still unclear. When we searched a possible binding site of miR-142-5p in the 3′-UTR of PSD-95, no site was identified from several target search algorithms (data not shown). Supporting this, there is no sequence motif (ACUUUAU) in the 3′-UTR of PSD-95 which is complementary to the seed sequence of miR-142-5p (AUAAAGU). This suggests that PSD-95 would be regulated by miR-142-5p indirectly. One possibility is that the suppression of miR-142-5p target genes may have decreased the expression of PSD-95. Several target genes shown in Figure 3C, including those for A-kinase anchor protein (AKAP5) and dopamine receptor D1 (DRD1), are known to interact with PSD-95. In neurons, AKAP5 directly interacts with PSD-95 at postsynaptic density, leading to the endocytosis of synaptic AMPA receptors (Colledge et al., 2000; Bhattacharyya et al., 2009). DRD1 interacts with the N-terminus of PSD-95 and maintains the balance of dopamine receptor-glutamatergic scaffold interaction (Zhang et al., 2007). Thus, miR-142-5p may affect PSD-95 indirectly by regulating the expression of other neuron-related genes. This necessitates further studies to gain insight in the detail mechanism of the effect of miR-142-5p during the pathogenesis of AD.

There need to be additional studies to use miR-142-5p as a therapeutic target of AD. One of the key experiments is to test whether the inhibition of miR-142-5p expression in the brain of animal AD model could restore the loss of PSD-95 and subsequently prevent neuronal loss and memory decline. Thus, the establishment of a proper method to manipulate the level of miR-142-5p would be important to verify the role of this miRNA in the regulation of synaptic plasticity and to alleviate the pathogenesis of AD.

## Author Contributions

JS conducted cellular experiments and wrote the preliminary draft. Y-KK performed bioinformatics analysis, revised the manuscript and provided overall supervision for the project.

## Conflict of Interest Statement

The authors declare that the research was conducted in the absence of any commercial or financial relationships that could be construed as a potential conflict of interest.

## Footnotes

^1^https://www.ncbi.nlm.nih.gov/geo/

^2^http://hannonlab.cshl.edu/fastx_toolkit/

^3^http://www.targetscan.org

^4^http://software.broadinstitute.org/gsea/msigdb
