# Supplementary material for: Identification of the Role of miR-142-5p in Alzheimer’s Disease by Comparative Bioinformatics and Cellular Analysis
Source: Front Mol Neurosci. 2017 Jul 18;10:227. doi: 10.3389/fnmol.2017.00227 (PMC5513939; doi:10.3389/fnmol.2017.00227)
Supplement: Supplementary file 1 [file Data_Sheet_1.docx]

**Supplementary materials**


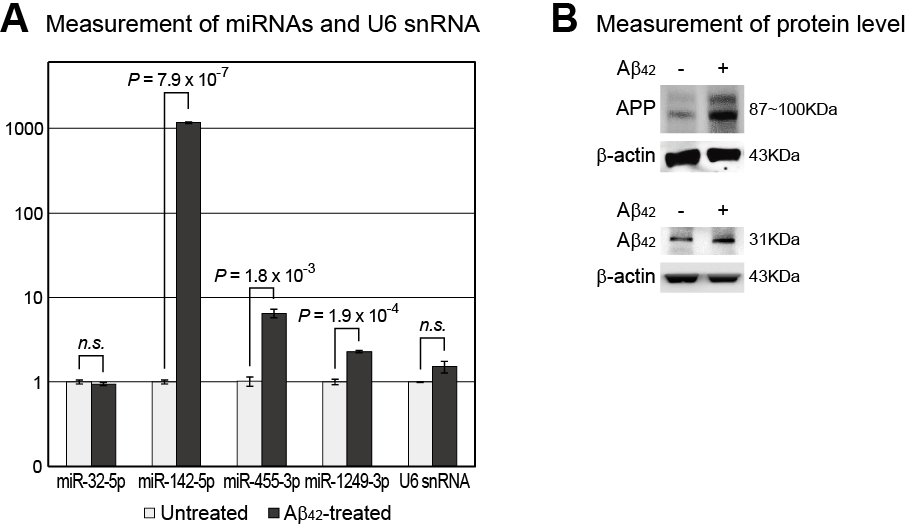


**Supplementary Figure 1. Measurement of miRNAs, U6 snRNA, and protein levels in a cellular AD model. (A)** Expression of miRNAs in Figure 1C without normalization against U6 snRNA. Expression level of each miRNA and U6 snRNA, respectively, is shown. The result shows that treatment of Aβ_42_ does not affect U6 snRNA level significantly. Data are presented as mean ± standard error (n=3). *P* values were calculated using two-tailed *t*-test. *n.s.* indicates not significant. **(B)** For the same cells in (A), we measured the expression change of amyloid precursor protein (APP), which verifies the effect of Aβ_42_ treatment.


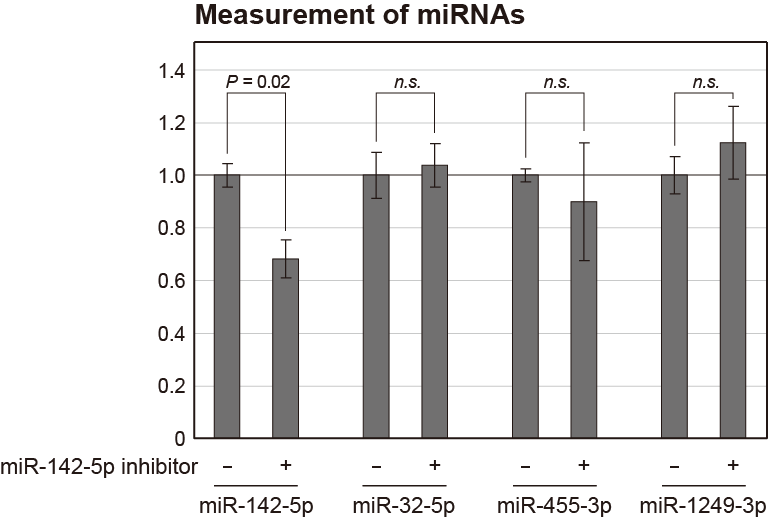


**Supplementary Figure 2. Validation of miR-142-5p inhibitor.** To validate the efficacy of miRNA inhibitor, we measured the levels of miR-142-5p and several other unrelated miRNAs in the control inhibitor- or miR-142-5p inhibitor-treated sample. The miR-142-5p inhibitor only suppressed miR-142-5p without affecting other unrelated miRNAs. Error bars indicate standard error (n=3). *P* values were calculated using two-tailed *t*-test. *n.s.* indicates not significant.

**Supplementary Table 1. Expression level change of miRNAs selected in Figure 1A.** Those miRNAs listed in **Figure 1B** are shown with bold letters.

| **miRNA name** | **Log_2_Fold (Human GSE48552)** | **Log_2_Fold (Human GSE63501)** | **Group (A)?** | **Group (B)?** | **Group (C)?** | **Log_2_Fold (Mouse APP/WT) in Group (C)** |
| --- | --- | --- | --- | --- | --- | --- |
| **miR-1249-3p** | -0.861 | -0.966 | Y |  | Y | -0.460 |
| **miR-132-5p** | -1.783 | -0.215 |  | Y | Y | 0.483 |
| **miR-142-5p** | 1.839 | 0.564 |  | Y | Y | 0.730 |
| **miR-150-5p** | -1.062 | 0.117 |  | Y | Y | 0.430 |
| **miR-153-3p** | 1.680 | -0.201 |  | Y | Y | -0.387 |
| **miR-210-3p** | -0.757 | -0.645 | Y |  | Y | 0.479 |
| **miR-212-5p** | -1.640 | 0.064 |  | Y | Y | -0.386 |
| **miR-29c-3p** | 0.189 | 1.053 |  | Y | Y | 0.400 |
| **miR-32-5p** | 1.248 | -0.031 |  | Y | Y | 0.575 |
| **miR-339-3p** | -1.070 | 0.026 |  | Y | Y | 0.601 |
| **miR-374b-5p** | 1.399 | 0.133 |  | Y | Y | 0.355 |
| **miR-423-3p** | -1.188 | -0.169 |  | Y | Y | 0.492 |
| **miR-431-3p** | -0.833 | -0.876 | Y |  | Y | 0.368 |
| **miR-431-5p** | -1.048 | -0.588 | Y |  | Y | 0.443 |
| **miR-455-3p** | 0.207 | -1.357 |  | Y | Y | -1.480 |
| **miR-487b-3p** | -1.029 | -0.085 |  | Y | Y | 0.357 |
| **miR-501-3p** | 1.405 | 0.110 |  | Y | Y | -0.975 |
| **miR-99a-5p** | 1.756 | 0.435 |  | Y | Y | -0.666 |
| miR-103a-1-3p | -1.114 | -1.121 | Y |  |  |  |
| miR-103a-2-3p | 0.605 | 2.134 | Y |  |  |  |
| miR-1185-5p | -0.722 | -0.836 | Y |  |  |  |
| miR-1224-5p | -0.787 | -0.635 | Y |  |  |  |
| miR-125b-1-3p | 1.314 | -0.241 |  | Y |  |  |
| miR-132-3p | -1.988 | -0.150 |  | Y |  |  |
| miR-136-3p | -0.056 | 1.323 |  | Y |  |  |
| miR-141-3p | 1.556 | 0.591 | Y |  |  |  |
| miR-146a-5p | 1.231 | 0.305 |  | Y |  |  |
| miR-152-3p | 1.780 | 0.162 |  | Y |  |  |
| miR-181a-3p | -0.299 | -1.030 |  | Y |  |  |
| miR-183-5p | 1.206 | -0.191 |  | Y |  |  |
| miR-194-5p | 1.039 | 0.207 |  | Y |  |  |
| miR-199a-3p | 0.016 | 2.389 |  | Y |  |  |
| miR-199a-5p | 1.175 | 0.096 |  | Y |  |  |
| miR-208b-3p | 1.726 | 1.314 | Y |  |  |  |
| miR-212-3p | -1.725 | -0.556 |  | Y |  |  |
| miR-219a-5p | -0.689 | -0.648 | Y |  |  |  |
| miR-223-3p | 1.123 | 0.167 |  | Y |  |  |
| miR-26b-3p | -1.227 | 0.009 |  | Y |  |  |
| miR-29a-3p | 0.743 | 0.616 | Y |  |  |  |
| miR-301a-3p | 1.127 | 0.123 |  | Y |  |  |
| miR-3117-3p | -0.435 | -1.254 |  | Y |  |  |
| miR-320b | -0.712 | -0.621 | Y |  |  |  |
| miR-323a-5p | -1.517 | -0.336 |  | Y |  |  |
| miR-330-5p | 1.138 | -0.307 |  | Y |  |  |
| miR-335-3p | 0.882 | 0.720 | Y |  |  |  |
| miR-337-5p | -0.073 | 1.167 |  | Y |  |  |
| miR-338-3p | 1.227 | -0.294 |  | Y |  |  |
| miR-340-5p | -0.895 | -1.248 | Y |  |  |  |
| miR-365a-3p | 1.414 | 0.133 |  | Y |  |  |
| miR-370-3p | -1.030 | 0.308 |  | Y |  |  |
| miR-375 | -0.714 | -0.833 | Y |  |  |  |
| miR-376a-5p | -1.584 | -0.474 |  | Y |  |  |
| miR-377-3p | 1.379 | 2.367 | Y |  |  |  |
| miR-410-3p | -0.095 | 1.111 |  | Y |  |  |
| miR-411-5p | -0.034 | -1.138 |  | Y |  |  |
| miR-433-5p | -1.214 | -0.587 | Y |  |  |  |
| miR-454-3p | 1.014 | 0.167 |  | Y |  |  |
| miR-485-5p | -1.081 | -0.476 |  | Y |  |  |
| miR-488-3p | 1.403 | -0.006 |  | Y |  |  |
| miR-516a-5p | 1.699 | -0.076 |  | Y |  |  |
| miR-6842-3p | -1.050 | -0.174 |  | Y |  |  |
| miR-708-5p | 0.953 | 0.617 | Y |  |  |  |
| miR-7-1-3p | 1.587 | 0.429 |  | Y |  |  |
| miR-885-3p | -2.211 | -0.103 |  | Y |  |  |
| miR-885-5p | -1.206 | -0.859 | Y |  |  |  |
| miR-887-3p | -0.643 | -0.644 | Y |  |  |  |
| miR-941 | 2.098 | 0.879 | Y |  |  |  |
